# Cryptococcal sepsis in small vessel vasculitis

**DOI:** 10.4103/0971-4065.70850

**Published:** 2010-07

**Authors:** S. Satish, R. Rajesh, S. Shashikala, G. Kurian, V. N. Unni

**Affiliations:** Department of Nephrology, Amrita Institute of Medical Sciences and Research Center, Kochi, Kerala, India; 1 Department of Microbiology, Amrita Institute of Medical Sciences and Research Center, Kochi, Kerala, India

**Keywords:** Cryptococcemia, cryptococcemia, Wegener’s granulomatosis, immunosupression

## Abstract

While meningoencephalitis due to cryptococcus is well known in immunocompromised patients, disseminated cryptococcosis and cryptococcemia is rare outside the setting of advanced HIV infection. We report a case of disseminated cryptococcosis occurring in a patient with Wegener’s granulomatosis on immunosuppressive medications.

## Introduction

*Cryptococcus neoformans*, an encapsulated yeast, is a typical opportunistic pathogen. The most common predisposing factor for cryptococcosis worldwide, is advanced HIV infection with CD4+ counts usually below 200/μL. The other important predisposing factors include immunosuppressive therapy and hepatic cirrhosis.[1] Meningoencephalitis is the most common clinical manifestation of cryptococcosis. Cryptococcemia is reported in only 10–30 % of patients with cryptococcal disease.[1] The majority of nonHIV patients with cryptococcal disease do not have Cryptococcemia.[2]

## Case Report

A 46-year-old female presented to our hospital with history of dyspnoea, cough and hemoptysis of one-week duration. She had oliguria for two days which rapidly worsened to anuria on admission. She had no history of macrohematuria, headache, epistaxis or previous nephritic or nephrotic illness. She gave a history of polyarthralgia, skin rashes and intermittent fever. On admission she was febrile, pale, and the blood pressure was 140/90 mm Hg and respiratory system examination revealed coarse crepitations on both sides. The other systems were normal. She had no obvious upper respiratory tract abnormalities or focal neurological deficits. Ear, nose and tongue (ENT) examination was normal. Investigations revealed: Hb 9.2 g%, total WBC count 13,400/mm^3^, platelet count 400,000/mm^3^, ESR 88 mm at 1st hour, blood urea 153 mg%, S. creatinine 8.5 mg%, and urinalysis showed 3+ protein, numerous RBCs and RBC casts. Blood sugars and liver functions were normal. ANA, anti ds DNA, ASO titre, and anti GBM antibodies were negative, Serum complements were normal, c ANCA was 111 U/ml (positive), while p ANCA was negative. X-ray chest revealed bilateral pulmonary nonhomogenous opacities and ultrasonogram of abdomen normal sized kidneys with increased echotexture. CT scan of paranasal sinuses was normal. Blood and sputum cultures did not reveal any growth.

**Figure 1:**
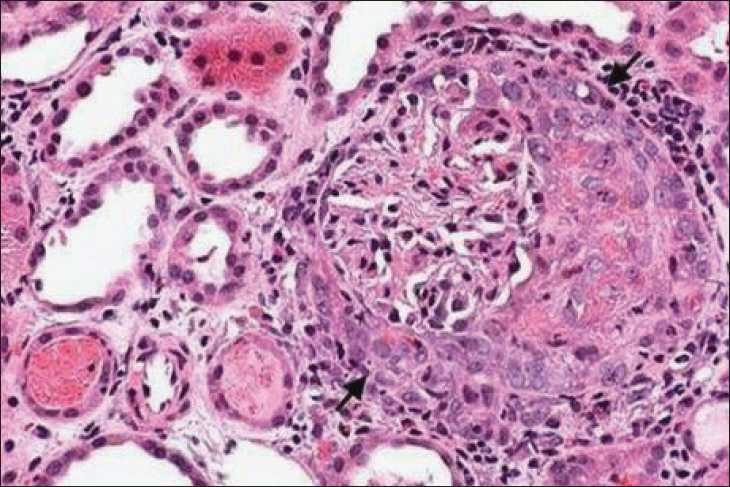
Kidney biopsy showing a glomerulus with a crescent (arrow) H and E stain, ×400

Renal biopsy [Figure 1] revealed 11 glomeruli, of which nine showed extra capillary proliferation with fibrocellular crescents and two showed cellular crescents; one glomerulus showed segmental necrotising glomerulonephritis. Interstitial oedema with mononuclear infiltrates was noted. Immunoflourescence showed no significant deposits. A diagnosis of Wegener’s granulomatosis was made, as she had a cANCA positive pauciimmune crescentic glomerulonephritis with evidence of lung involvement.

The patient was started on hemodialysis and was given intravenous Methylprednisolone 1 gram daily for 3 days followed by oral prednisolone 1 mg/kg daily. She was also started on Cyclophosphamide 2 mg/kg orally. She improved symptomatically. The urine output improved, dialysis was discontinued; the serum creatinine was 1.9 mg % on discharge, two weeks after admission. The opacities on X-ray chest improved.

**Figure 2:**
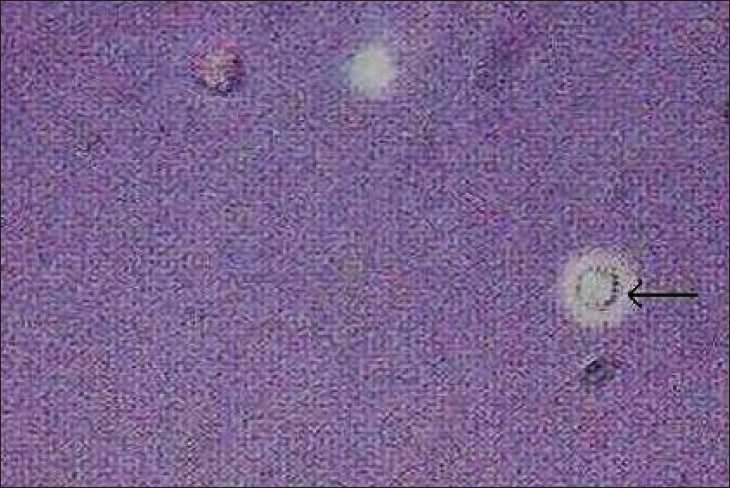
India ink stain of CSF showing cryptococci (arrow)

**Figure 3:**
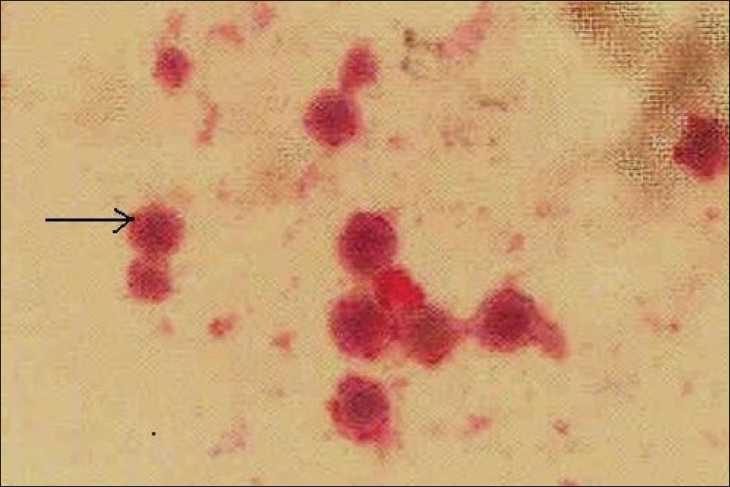
Blood culture showing cryptococci (Mucicarmine stain)

Unfortunately the patient stopped treatment and follow-up. She presented nearly ten months later to another hospital with fever and arthralgias, and was restarted on prednisolone and cyclophosphamide. Subsequently she presented to our centre after two months with fever, nausea and headache of one-week duration. She was found to have severe renal failure and was started on hemodialysis X-ray chest revealed bilateral nonhomogenous opacities. The fever and intense headache continued. MRI scan of brain was normal. Cerebrospinal fluid (CSF) study revealed raised proteins, low glucose, lymphocytic pleocytosis and India ink staining showed encapsulated yeast [Figure 2]. Test for capsular antigen of Cryptococcus in CSF was positive. Serology for HIV was negative. Blood, CSF and sputum culture grew cryptococci [Figure 3], which was identified as *C. neoformans*. Species identification was based on the biochemical characteristics (urease production and inositol assimilation). No bacteria were isolated from blood, sputum, CSF and urine. She was diagnosed to have disseminated Cryptococcosis. She was started on Amphotericin B. Cyclophosphamide was stopped and the dose of steroids reduced. She developed respiratory distress after five days necessitating ventilatory support. The patient deteriorated and expired nearly two weeks after admission.

## Discussion

*C. neoformans*, an encapsulated yeast found worldwide is an important opportunistic pathogen causing chronic meningitis in immunocompromised patients. *C. neoformans* is thought to gain access to the host by inhalation in most patients. The pulmonary infection is mostly asymptomatic and often undergoes spontaneous resolution. Depending on the immune status of the host, spores may remain dormant in the lungs or spread to other organs through blood. Hematogenous spread to brain may lead to meningoencephalitis.[5]

*C. neoformans* has a number of virulence factors that enable it to survive and replicate in the human host, especially if specific T-cell immunity is compromised: the ability to grow at 37°C, the capsule, which is antiphagocytic and down-regulates cellular and humoral immune responses when shed into host tissues, laccase and melanin that interfere with oxidative killing by phagocytes.[4] Production of melanin from levo dopa by the enzyme laccase may account for the predilection of the organism for the central nervous system (CNS). HIV infection, lymphoma, sarcoidosis, cirrhosis and idiopathic CD4 + lymphocytopenia predispose to Cryptococcal infection.[5] The source from which humans acquire the infection is unknown (an exception being transmission through transplanted organs). This fungal infection is uncommon before puberty[5]. Cyclosporine A has an anti cryptococcal effect as it inhibits fungal calcineurin. Hence this drug may provide some protective effect against *C. neoformans.*

Similar to a number of other chronic fungal and bacterial infections, protection is associated with an active granulomatous inflammatory response, and depends on intact cell-mediated immunity involving both CD4+ and CD8+ cells, and a Th1 pattern of cytokine release. Protective roles for tumour necrosis factor-α (TNF-α), interleukins 12 and 18 (IL-12, IL-18) and interferon-γ (IFN-γ) have been inferred from experiments with knockout mice and antibody neutralization.[4]

The most common clinical manifestation is meningoencephalitis, which is invariably fatal without appropriate treatment. Headache, fever, nausea, irritability and confusion are the common symptoms. Cranial nerve palsies, papilledema optic atrophy and central scotoma have been described. Pulmonary cryptococcosis presents with fever, cough and chest pain; dense or non-homogenous lesions may be seen on radiographs. Around 10 % of patients have skin lesions (papular lesions which enlarge slowly and show central softening or ulceration), which would show Cryptococci on biopsy. Rare manifestations include prostatitis, endophthalmitis, pericarditis, endocarditis, renal abscess[5] and tenosynovitis.[6] Previous studies have shown that cryptococcemia signifies a fulminant form of cryptococcal disease, and requires early diagnosis and prompt antifungal therapy.[1] In contrast to meningitic presentation, disseminated cryptococcosis can present as fulminant sepsis, and needs a high index of suspicion for diagnosis.

Laboratory diagnosis in suspected cryptococcal meningitis depends on CSF examination. The CSF white cell count is raised, with a predominance of lymphocytes, in non-HIV-associated infection. In HIV-associated cryptococcal meningitis the CSF white cell count is lower and may even be normal. CSF protein is usually elevated and CSF glucose may be low. India ink examination is positive in 70–90% of AIDS patients but in only about 50% of non-AIDS patients.[4] Cryptococci in tissues can be seen by Silver methenamine, Periodic acid Schiff or mucicarmine staining.

Other diagnostic modalities include testing for the capsular antigen in CSF or serum by latex agglutination. Detection of the cryptococcal polysaccharide antigen in body fluids by rapid and simple latex agglutination tests or enzyme immunoassay has a sensitivity >90% and, at a titre of >1:4, is very specific. Antibodies to *C. neoformans* are not useful in diagnosis. Fungemia has been reported in the majority of patients with AIDS (60%) compared with only 10–30 % in non-HIV patients.[5] *C. neoformans* from CSF, blood or other sites produces white mucoid (depending on the capsule thickness) colonies, usually within 48–72 h, on most bacterial and fungal (Sabouraud dextrose agar) media at 20–37°C. Although *C. neoformans* grows at 37°C, a temperature of 30–35°C is optimal. Standard blood culture systems will detect cryptococcemia. Identification is based on culture characteristics, microscopic appearance, biochemical tests, such as urease production, or DNA-based methods.[5]

Amphotericin B is the mainstay of treatment for all forms of cryptococcosis. In patients with AIDS, current guidelines recommend initial two weeks of Amphotericin B 0.7–1 mg/kg/day. Addition of flucytosine 100 mg/kg/day may provide additional benefit. Subsequently fluconazole 400 mg/day for next 8 weeks and 200 mg/day indefinitely till immune reconstitution (in AIDS patients) is recommended.[4] In patient without AIDS, the aim of treatment is to cure Cryptococcal meningitis. Therapy with Amphotericin B for at least 10 weeks is warranted and all previously positive cultures should become negative. Normalization of CSF glucose and fall in antigen titres mark successful treatment. Following Amphotericin, Fluconazole may be given for six months.[4]

The poor prognostic factors in nonHIV patients including female, age more than 60 years, severe underlying illness, high Cryptococcal antigen titres, large number of cryptococci in CSF and severe sepsis syndrome. The mortality rates of cryptococcal infection has been reported to be between 10 and 48 % in various studies.[1,3] In cases with cryptococcosis and disseminated infection, the 30 day mortality is 37 %.[1]

This patient had cryptococcal sepsis in a nonAIDS setting. As Cryptococci were isolated from CSF, sputum and blood, she had disseminated Cryptococcosis. The severity of the sepsis as well as the underlying immunosupression probably contributed to the poor response to antifungal therapy and a fatal outcome. This report highlights the need to consider Cryptococci as a cause of sepsis syndrome in immunocompromised patients, especially if bacteria are not isolated.
